# Motivation to participate in high-intensity functional exercise compared with a social activity in older people with dementia in nursing homes

**DOI:** 10.1371/journal.pone.0206899

**Published:** 2018-11-14

**Authors:** Anna Sondell, Erik Rosendahl, Johan Nilsson Sommar, Håkan Littbrand, Lillemor Lundin-Olsson, Nina Lindelöf

**Affiliations:** 1 Department of Community Medicine and Rehabilitation, Physiotherapy, Umeå University, Umeå, Sweden; 2 Department of Community Medicine and Rehabilitation, Geriatric Medicine, Umeå University, Umeå, Sweden; 3 Department of Public Health and Clinical Medicine, Occupational and Environmental Medicine, Umeå University, Umeå, Sweden; Johns Hopkins University Bloomberg School of Public Health, UNITED STATES

## Abstract

**Background:**

Motivation to participate in exercise among people with dementia has not been well studied. The symptoms of dementia, including apathy, may lead to low motivation to participate in exercise. The aim of this study was to evaluate the motivation of older people with dementia to participate in a high-intensity exercise program compared with motivation of those participating in a social group activity.

**Methods:**

The Umeå Dementia and Exercise Study (UMDEX) was a cluster-randomized controlled intervention trial including 186 people (mean age; 85, 75% female) with dementia in nursing homes. Participants were randomized to participate in the High-Intensity Functional Exercise (HIFE) Program (n = 93) or a seated social group activity (n = 93). The activities were conducted in groups of 3–8 participants for 45 minutes, five times per two-week period, for 4 months (40 sessions in total). Participants’ motivation to go to and during activity sessions were assessed by the activity leaders and nursing homes staff using a five-point Likert scale. Data were analyzed using cumulative link mixed models.

**Results:**

Motivation was high or very high during 61.0% of attended sessions in the exercise group and 62.6% in the social activity group. No overall significant difference between groups was observed, but motivation increased over time in the exercise group and decreased in the social activity group (*p* < 0.05). Motivation during the sessions was significantly higher than motivation to go to the sessions, especially in the exercise group [OR 2.39 (95% CI 2.38–2.40) and 1.50 (95% CI 1.32–1.70), respectively].

**Conclusions:**

Among older people with dementia in nursing homes, motivation to participate in a high-intensity functional exercise program seems to be high, comparable to motivation to participate in a social activity, and increase over time. Since motivation during activity sessions was higher than motivation to go to sessions the promotion of strategies to encourage people with dementia to join exercise groups is of great importance.

## Introduction

People with dementia are in need of rehabilitation, including physical exercise, because of successive deterioration of cognitive and physical function [1–5], associated with loss of independence in activities of daily living (ADLs) [6–8]. In older people worldwide, dementia is the leading cause of dependency in ADLs [9]. Promising results, however, suggest that physical exercise can slow declines leading to ADL dependency [10–12]; improve physical functioning, such as muscle strength, balance, and gait [12–15]; and reduce falls [16] in people with dementia. Recommendations indicate that exercise should be task specific, performed at high intensity, i.e., near the individual`s maximum capacity [17–20], and carried out at sufficient duration and frequency (2–3 times per week for balance and resistance exercise) [17,21] to have optimal effects in older people. Motivation to participate in an exercise program is an important aspect of fulfilling these recommendations because it affects attendance and the exercise intensity achieved [22,23], and thereby the overall effect.

Motivation concerns energy, direction and persistence, all aspects of activation and intention [24,25]. In the self-determination theory (SDT) [25], motivation requires autonomy, competence, and relatedness for optimal functioning [26], which can be applied to different fields, including physical activity. Self-efficacy is associated with motivation, and is an important aspect of social cognitive theory; and it concerns the belief in one's ability to succeed in specific situations or accomplish a task [27]. Older people with dementia are at particular risk of low motivation for activities for several reasons. People with dementia form a heterogeneous group with a range of cognitive and physical impairments, including behavioral and psychological symptoms of dementia (BPSDs), depressive symptoms, and other comorbidities, which can vary over time and negatively impact the motivation to participate in exercise [9]. Apathy, or lack of motivation and interest in activities, affects more than 70% of people with dementia [28,29]. In addition, the motivation of people in more advanced stages of dementia to participate in rehabilitation may be challenging because some of these people live mostly in the present moment [29], which can reduce insight into their needs and consideration of future gains afforded by exercise. Anxiety about upcoming events is also common in this group [30], and can negatively affect interest in joining exercise sessions. There are several types of physical activity interventions that have been performed in people with dementia [31–33]. Little is known about which interventions suit particular needs and preferences in people with dementia [34]. Knowledge about motivation is important for the facilitation of exercise participation in this population. Individuals with cognitive impairment have expressed preferences for simple, light, and safe exercise [35]. Thus, people with dementia might be more motivated to participate in a social activity than in high-intensity exercise.

A review of barriers, motivators, and facilitators related to physical activity in people with dementia demonstrated the importance of considering how a program can be personalized to and synchronized with individuals’ needs, leading to a call for further studies of the matter among institutionalized people with dementia [36]. Previous studies of motivation to exercise in people with cognitive impairment have focused on exercise preferences [35], perceptions about exercise [37], and motivators applicable in different exercise scenarios [36]. To our knowledge, no quantitative study has investigated motivation while participating in any type of exercise, including high-intensity functional exercise, among people with dementia. This type of exercise seems to be the most effective but, at the same time, a demanding type of exercise [17,18].

The aim of this study was to evaluate the motivation of older people with dementia to participate in a high-intensity functional exercise program compared with participation in a social group activity. More precisely, we evaluated motivation to go to the activity and motivation during the activity, as well as variation in motivation over the 4-month intervention period.

## Methods

This study was part of the Umeå Dementia and Exercise Study (UMDEX), a cluster-randomized controlled trial examining the effects of a high intensity exercise program among people with dementia living in nursing homes. The intervention was conducted in the municipality of Umeå, Sweden, in 2011 and 2012. UMDEX has been described in detail elsewhere [12]. The study protocol (ISRCTN31767087) is published in the ISRCTN registry. The Regional Ethical Review Board of Umeå approved the study (2011-205-31M).

### Settings and participants

Nursing homes with 30 residents or more within the municipally of Umeå were eligible. The 16 nursing homes included, comprised 9 general and 10 dementia units, all with private rooms and staff on hand, as well as private apartments with access to on-site nursing and care. A total of 186 participants were included in the study. Inclusion criteria were a diagnosis of dementia according to the Diagnostic and Statistical Manual of Mental Disorders, Fourth Edition, Text Revision (DSM-IV-TR), age ≥ 65 years, dependency in personal ADLs according to the Katz Index [38], ability to stand up from a chair with armrests with help from no more than one person, a Mini-Mental State Examination (MMSE) score ≥ 10 [39], ability to hear and understand the Swedish language sufficiently well to participate in assessments, and approval from the resident´s physician. Included participants gave informed oral consent to participate, which was affirmed orally by their next of kin.

### Randomization

Two researchers not involved in the study performed randomization after completion of the inclusion process and baseline assessment, to ensure that allocation was concealed and to avoid selection bias. To avoid contamination between activities, clusters were formed comprising 3–8 inhabitants of the same wing, unit or floor. Randomization of the 36 clusters was stratified to ensure that clusters of both interventions were present in each facility, reducing the risk that factors associated to the facility would influence the outcome. Randomization was performed by drawing lots in sealed opaque envelopes; the order in which clusters were allocated was determined first, followed by random allocation of participants to the exercise and social activity groups [12].

### Intervention

Both activities, i.e. exercise and social activity, were conducted at the nursing homes in groups of three to eight participants, supervised by two physical therapists (PT) or one occupational therapist (OT)/OT assistant, all experienced in working with older people with cognitive impairment. Sessions of approximately 45 minutes were held five times per two-week period, for 4 months, with a total of 40 sessions held for each group. The activity leaders obtained updates on participants’ health status before the sessions and were able to contact physicians or nurses when necessary. At the end of each activity session, activity leaders completed a structured protocol for each participant including reasons for non-attendance. Before starting each activity session, the activity leaders or nursing home staff gave verbal reminders or aided transfer to the session, and also when needed, motivated the participants to join the activity session.

### Exercise program

The exercise intervention was based on the High-Intensity Functional Exercise (HIFE) Program developed by members of the research team [22], available as a booklet [40] and on a webpage; https://www.hifeprogram.se/en [41]. The program is designed to improve lower limb strength, balance, and mobility in older people with various levels of physical impairments, by high-intensity functional exercises. The program comprises 39 exercises to be performed in functional weight-bearing positions similar to those used in everyday situations, such as rising from a chair, step-ups, trunk rotation, walking, and climbing stairs [40]. The exercises are distributed over 5 categories: A, static and dynamic balance exercises in combination with lower-limb strength exercises; B, dynamic balance exercises in walking; C, static and dynamic balance exercises in standing; D, lower-limb strength exercises with continuous balance support; and E, walking with continuous balance support. In order to start the exercise period on an appropriate level, exercises are chosen according to a hierarchical model based on level of support required while walking a short distance (5–10 m). Exercises were chosen for each participant depending on the degree of functional deficit, and adapted individually throughout the intervention period to meet the cognitive deficit level, BPSDs, and changes in health and functional status. The intensity of strength and balance exercises was intended to be high, with progression through increased load and difficulty. Participants were supervised individually to promote the highest possible exercise intensity while ensuring their safety. High-intensity strength exercises were performed at 8–12 repetition maximum (RM). The load was increased by adjusting performance of the exercise or by using a weighted belt, loaded with a maximum of 12 kg. The balance exercises were intended to fully challenge participants’ postural stability and progressed by, for example, narrowing the base of support or altering the surface. Each of the two physiotherapists was responsible for the same participants (n = 1–4 per group), all with an individual exercise program prepared in advance.

Each group session started with joint warmup exercises in sitting for all participants. Participants were supervised individually to safely promote the highest possible exercise intensity. Participants took turns exercising and resting during each session. For safety, participants wore belts with handles so that PTs could provide support if needed when exercising near the limits of postural stability, thereby preventing falls. After each session, PTs estimated the exercise intensity achieved by each participant (high, medium, or low) according to a predefined scale [22]. Strength exercises were performed at moderate to high intensity at a median of 94.7% of attended sessions and balance exercises were performed at high intensity at a median of 75.0% of attended sessions [42].

### Social activity

The OT and OT assistant who took part in the study developed the social group activity. The sessions were structured around topics believed to be of interest to older people with dementia, such as, seasons and holidays, wildlife, gardening, the royal family, bakery, leisure activities, crafts, and well-known authors and artists. While sitting together in a group, participants engaged in activities including conversation, singing, listening to music or poetry, and looking at pictures and objects associated with the topic, for example newspapers, books, spices, plants and crafts. The activity leader had an active role in sharing own experiences and paying attention to and encouraging every individual. The participants were encouraged to answer questions freely. The interaction was also facilitated by, for example, the use of nametags. The activity did not involve physical activity.

### Outcome measures

The activity supervisors assessed each participant’s motivation during each session (n = 40), based on their observations and interpretations of participants’ expressions, verbal prompts, and body language. Each participant’s eagerness to participate was classified using a five-point Likert scale [0, no motivation (is present without participating); 1, low motivation (needs to be convinced to participate); 2, moderate motivation (attends the activity without being positive or negative); 3, high motivation (participates positively); 4, very high motivation (participates very positively)]. The motivation scales were based on the Eagerness scale, which has been developed and used in studies in similar settings and participants [43].

Motivation to go to activity sessions was assessed 4 times (A, B, C, D) over the intervention period, by the persons who most often helped the participant to the sessions (facility staff or activity leader–who sometimes varied over the intervention period). At each time the previous 2 weeks´ average motivation to go to the activity was assessed; [sessions 3–7 (A), 13–17 (B), 23–27 (C), and 31–35 (D)] using a five-point Likert scale [0, very negative (never wants to or usually does not want to go to sessions, despite motivating attempts); 1, negative (needs to be motivated to go to sessions, which s/he usually does); 2, neither positive nor negative (goes to sessions without being motivated); 3, positive; 4, very positive].

### Baseline measures

PTs and physicians performed all baseline assessments before randomization. ADL status was assessed using the Barthel ADL Index [44]. Functional balance capacity was assessed with the Berg Balance Scale (BBS) [45]. Usual gait speed was measured over 4 m with use of participants’ ordinary walking aids (when applicable). Depressive symptoms were assessed using the 15-item version of the Geriatric Depression Scale [46], global cognitive function was assessed using the MMSE [39], BPSDs were assessed using the Neuropsychiatric Inventory [47], and nutritional status was assessed using the Mini Nutritional Assessment [48]. Participants were also asked questions about their perceived loneliness and general health. Diagnoses based on information gathered from assessments, medical records, and medication prescriptions were recorded. Physicians specialized in geriatric medicine diagnosed dementia type according to the DSM-IV TR [49].

### Statistical analyses

Baseline characteristics were compared between the exercise and social activity groups using Student´s *t*-test and the chi-squared test. These analyses were performed using SPSS software (version 21.0; IBM Corporation, Armonk, NY, USA) with a two-tailed significance level of *p* < 0.05.

Motivation to go to activity sessions and motivation during the sessions were modelled using cumulative link mixed models (CLMM) with individual as random effect, where results were reported as the odds ratio (OR) of motivation being rated above any category comparing different sets of covariate values. The estimates were expressed as cumulative ORs, i.e., the odds ratio of motivation > 0 vs. 0, >1 vs. ≤1, >2 vs. ≤2, >3 vs. ≤3, and >4 vs. ≤4. This means that these odds of higher motivation were assumed to be proportional when comparing the exercise group with the social activity group. This assumption was evaluated by relaxing it and performing comparisons based on the Akaike information criterion. Motivation to go to activity was compared with motivation during activity by modelling these motivation assessments in relation to an indicator variable for motivation during activity. Confidence intervals (CIs) were based on 1000 bootstrap samples, where bootstrap samples were sampled with replacement. These analyses were done using R version 3.2.3 (R Development Core Team, 2010)[50].

Descriptive statistics were used to compare motivation during attended sessions and motivation to go to sessions in assessment periods A–D (20 sessions in total for each participant, i.e. 3720 possible comparisons in total sample). Individual differences for each session were categorized as lesser, equivalent, or greater motivation during the session than before it. In total there were 2551 comparisons (n = 1235 exercise, n = 1316 social activity). Missing data was mainly due to non-attendance. Investigation of selection bias due to loss of participants is presented in S1 Appendix.

## Results

**Fig 1:**
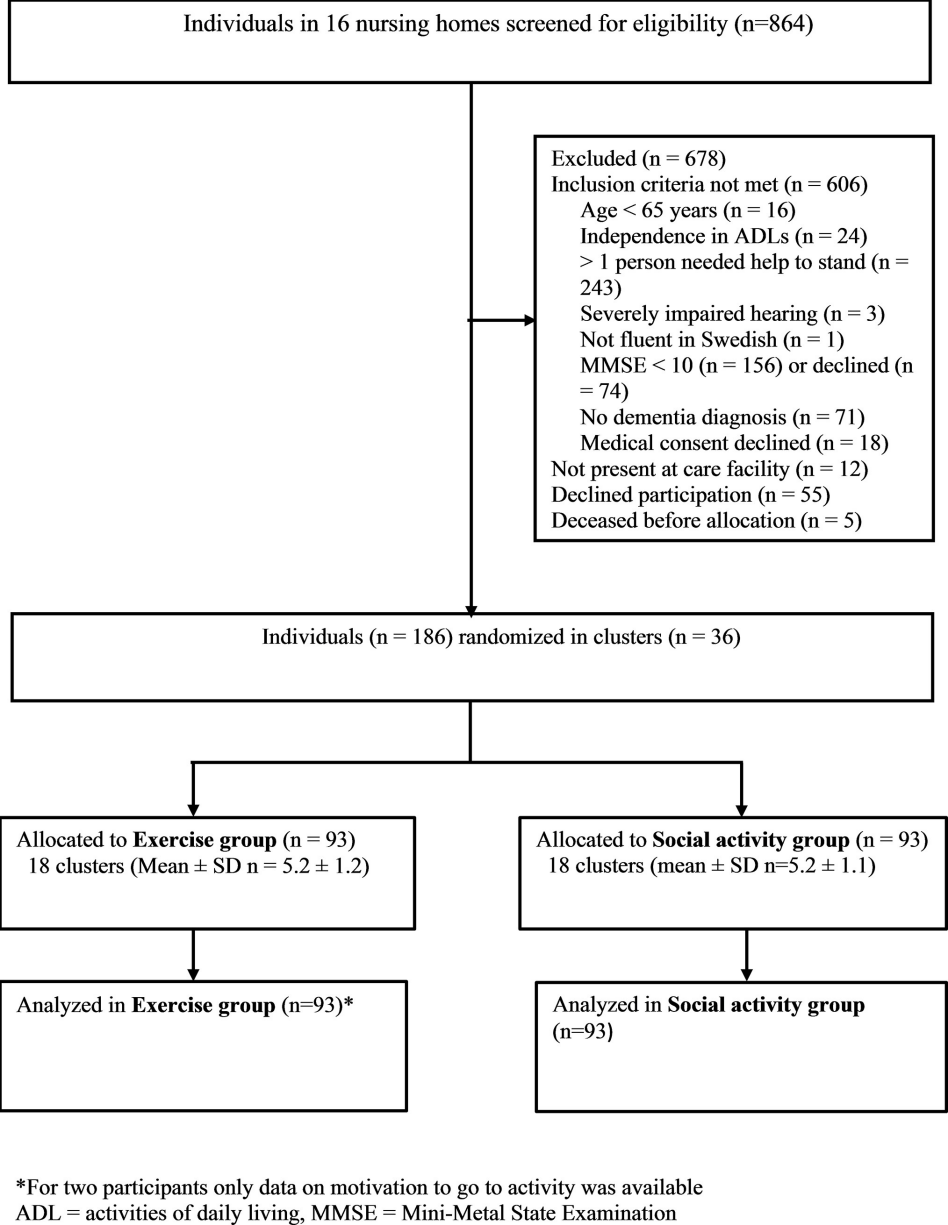
Flowchart of study participation.

In total, 186 participants (141 women and 45 men) were included in the study; 93 participants were allocated to each group (Fig 1). The mean (± standard deviation) MMSE score was 14.9 ± 3.5. Fifty-eight percent of participants had depression and 21% had apathy symptoms. Among baseline characteristics, only antidepressant use differed significantly between groups (Table 1). Attendance rates were 73.4% (2729 of 3720 possible sessions) in the exercise group and 69.5% (2587 of 3720 possible sessions) in the social activity group.

**Table 1:** Baseline characteristics of participants.

Characteristics	Total n = 186	Exercise n = 93	Social activity n = 93
Age	85.1 ± 7.1	84.4 ± 6.2	85.9 ± 7.8
Female	141 (75.8)	70 (75.3)	71 (76.3)
Previous exerciser	135 (72.6)	72 (77.4)	63 (67.7)
*Dementia type*:			
Alzheimer	67 (36.0)	34 (36.6)	33 (35.5)
Vascular	77 (41.4)	36 (38.7)	41 (44.1)
Other	27 (14.5)	15 (16.1)	12 (12.9)
Mixed Alzheimer/vascular	15 (8.1)	8 (8.6)	7 (7.5)
*Diagnoses and medical conditions*			
Depressive disorders	107 (57.5)	53 (57)	54 (58.1)
Delirium, previous week	102 (54.8)	48 (51.6)	54 (58.1)
Previous stroke	57 (30.6)	33 (35.5)	24 (25.8)
Heart failure	56 (30.1)	24 (25.8)	32 (24.4)
Previous hip fracture	53 (28.5)	28 (30.1)	25 (26.9)
Angina pectoris	49 (26.3)	21 (22.6)	28 (30.1)
Diabetes mellitus	29 (15.6)	18 (19.4)	11 (11.8)
Rheumatic disease	28 (15.1)	14 (15.1)	14 (15.1)
Chronic lung disease	39 (21.0)	20 (21.5)	19 (20.4)
Osteoarthritis	61 (32.8)	35 (37.6)	26 (28.0)
Vision impairment	26 (14.0)	10 (10.8)	16 (17.2)
Hearing impairment	32 (17.2)	12 (12.9)	20 (21.5)
*Prescribed medications*			
Analgesics	112 (60.2)	55 (59.1)	57 (61.3)
Antidepressants	102 (54.8)	58 (62.4)	44 (47.3)*
Benzodiazepine	40 (21.5)	19 (20.4)	21 (22.6)
Diuretics	88 (47.3)	41 (44.1)	47 (50.5)
Anti-dementia drugs	47 (25.3)	28 (30.1)	19 (20.4)
Number of medications	8.3 ± 3.8	8.4 ± 4.0	8.2 ± 3.7
*Assessments*			
MMSE (range 0–30)	14.9 ± 3.5	15.4 ± 3.4	14.4 ± 3.5
Barthel ADL Index (range 0–20)	10.8 ± 4.4	10.7 ± 4.5	11.0 ± 4.4
BBS (range 0–56)	28.9 ± 14.5	28.6 ± 14.3	29.3 ± 14.7
Gait speed over 4 m, m/s, n = 185	0.45 ± 0.2	0.45 ± 0.2	0.45 ± 0.2
NPI (range 0–144)	14.8 ± 3.2	15.2 ± 15.8	14.4 ± 12.6
GDS -15 (range 0–15), n = 185	3.8 ± 3.2	4.0 ± 3.4	3.6 ± 2.9
MNA (range 0–30), n = 185	21.1 ± 2.7	21.3 ± 2.8	20.9 ± 2.6
Self-reported good health	119 (64.0)	60 (64.5)	59 (63.4)
Perceived loneliness	104 (55.9)	50 (53.8)	54 (58.1)

Values are expressed as n (%) or mean ± standard deviation. Numbers reported after assessment measures indicate numbers of measures available when values were missing. For the BBS, MMSE, Barthel ADL Index, and MNA, higher scores indicate better status. For the GDS-15 and NPI, lower scores indicate better status.

*p = 0.04, difference between groups.

MMSE = Mini-Mental State Examination; ADL = activities of daily living; BBS = Berg Balance Scale; NPI = Neuropsychiatric Inventory; GDS-15 = 15-item Geriatric Depression Scale; MNA = Mini Nutritional Assessment.

### Motivation during activity sessions

**Fig 2:**
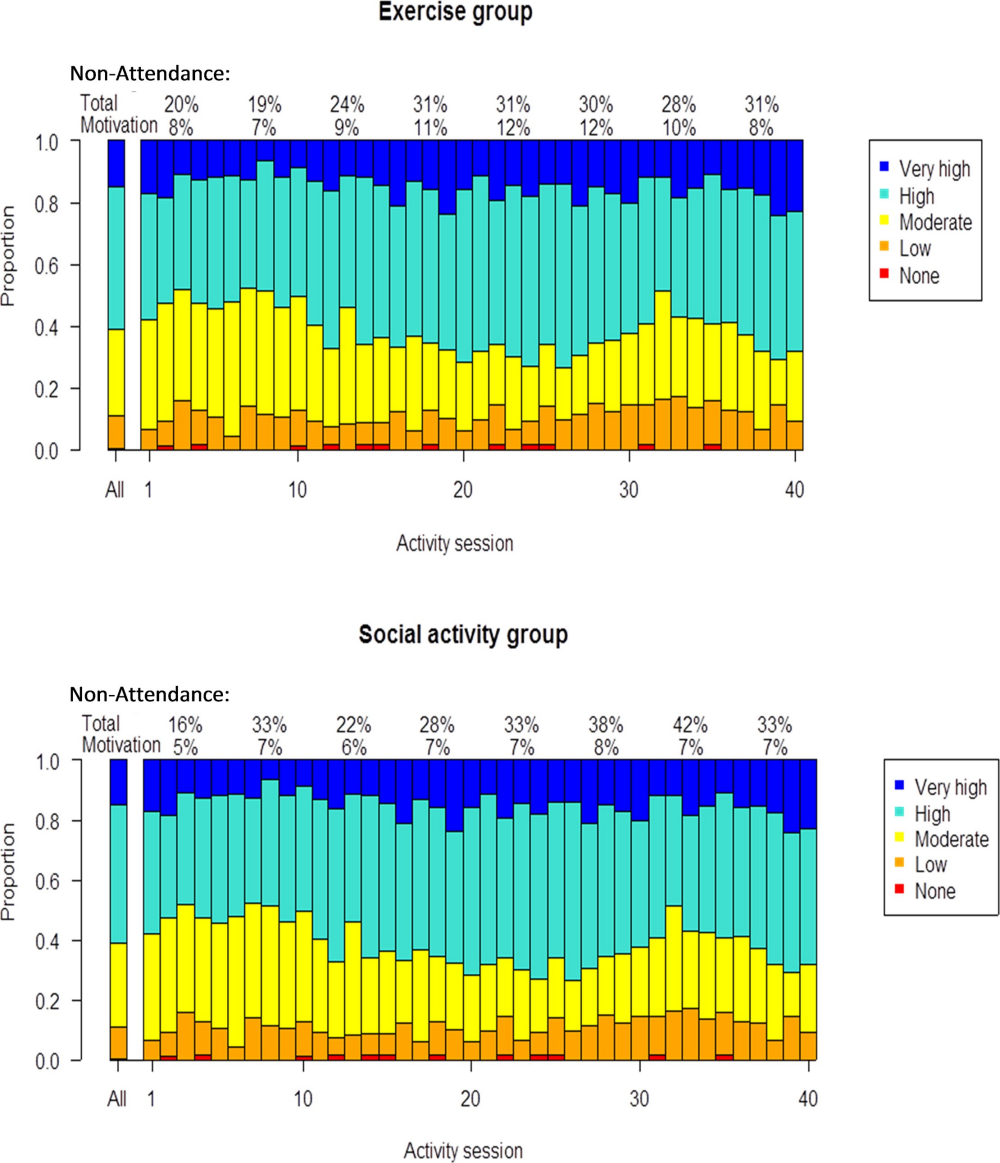
Motivation during activity sessions. Proportion of motivation scores during activity sessions, in total and for each session. The subheading of the figure presents mean average non-attendance in total and mean average non-attendance due to low motivation, calculated over five consecutive sessions.

High or very high motivation was recorded in the exercise group for 61.0% (1662/2726) of attended sessions, and in the social activity group for 62.6% (1618/2586) of attended sessions (Fig 2). Corresponding figures for no or low motivation during sessions were 11.5% in the exercise group and 10.0% in the social activity group.

**Fig 3:**
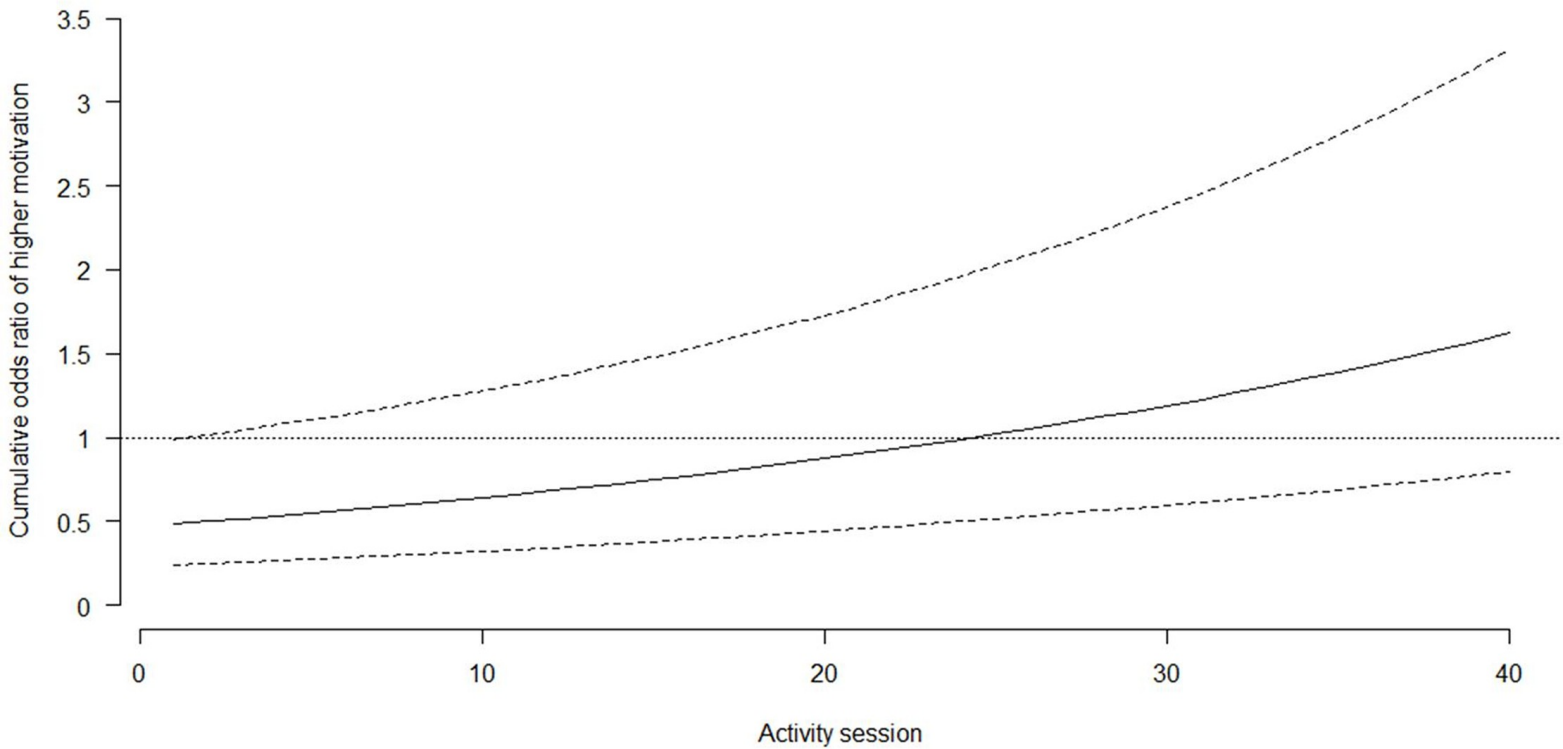
Comparing motivation during activity session in exercise group (solid line) relative to the social activity group (horizontal line 1.0). Dotted lines indicates the 95% confidence interval.

Motivation during sessions did not differ significantly between groups. On average, during the 40 sessions, the cumulative OR for greater motivation in the exercise group relative to the social activity group was 0.86 (95% CI 0.44–1.70). These odds were found to be constant over categories of motivation. During the course of the intervention period, linear trends in average motivation during activities were found. The cumulative odds of greater motivation during each subsequent activity session increased in the exercise group (OR 1.01, 95% CI 1.01–1.02), but decreased in the social activity group (OR 0.98, 95% CI 0.97–0.99). These time trends differed significantly, and the additional cumulative OR in the exercise group compared with the social activity group was 1.03 (95% CI 1.02–1.04; Fig 3). The cumulative odds of a higher motivation score at the first activity session was 51% (95% CI 2–76%) lower in the exercise group than in the social activity group (Fig 3).

Evaluation of variations in scoring (increasing or decreasing on the Likert scale) of motivation scores during activity sessions were evaluated by comparing motivation during activity between one activity sessions to the next. In the exercise group, 67% of all activity motivation scores were the same as during the previous session, 28% were single-category changes, 5% were two-category score changes, and 0.6% were three-category score changes. Corresponding proportions in the social activity group were 58%, 38%, 4%, and 0.4%, respectively. Forty percent of the attending participants in the exercise group and 38% in the social activity group showed at least one score change in motivation of two categories or more between consecutive sessions. Among such participants in the exercise group, this degree of score change in motivation occurred once for 13%, twice for 10%, three times for 4%, and 5–10 times for 13% of participants.

### Motivation to go to activity sessions

**Fig 4:**
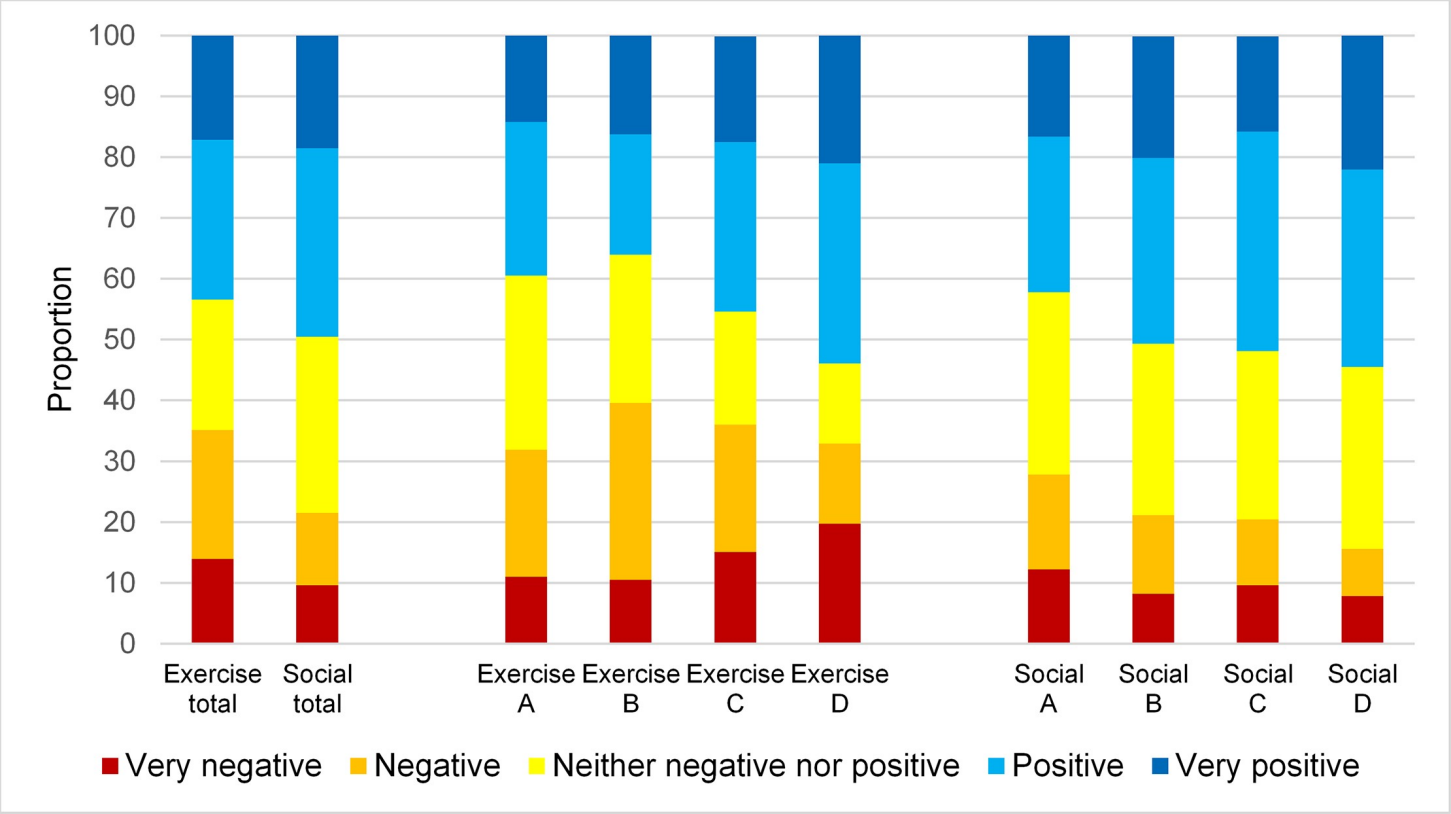
Motivation to go to activity sessions in exercise and social activity group, in total and in assessment periods A-D.

For all participants and assessment periods, positive or very positive motivation to go to sessions were noted in 43.4% of cases in the exercise group and 49.5% of cases in the social activity group. Corresponding figures for negative or very negative motivation to go to sessions were 35.1% in the exercise group and 21.5% in the social activity group. Fig 4 shows the distribution of ratings of motivation to go to sessions in total and for periods A–D in the exercise and the social activity groups.

On average, the cumulative odds of greater motivation was significantly lower in the exercise group than in the social activity group (OR 0.71, 95% CI 0.63–0.80). The cumulative odds of greater motivation increased with time, but less so in the exercise group. The cumulative OR for greater motivation to go to sessions in the exercise group relative to the social activity group in period A was 0.79 (95% CI 0.59–1.41). Thereafter, these odds first decreased by 13% (95% CI -26–38%) and then increased by 24% (95% CI -13–84%) and 84% (95% CI 21–179%). The cumulative odds of higher motivation increased in the social activity group by 65% (95% CI 18–123%), 39% (95% CI 1–90%), and 130% (95% CI 59–215%) in assessment periods B–D, respectively. Cumulative ORs for differences in greater motivation to go to sessions were higher in the social activity group and differed significantly between groups in all assessment periods (*p* < 0.001). The cumulative odds were 55, 25, and 32% lower in the exercise group compared with the social activity group for period B, C and D, respectively.

### Motivation during activity sessions compared with motivation to go to activity sessions

**Fig 5:**
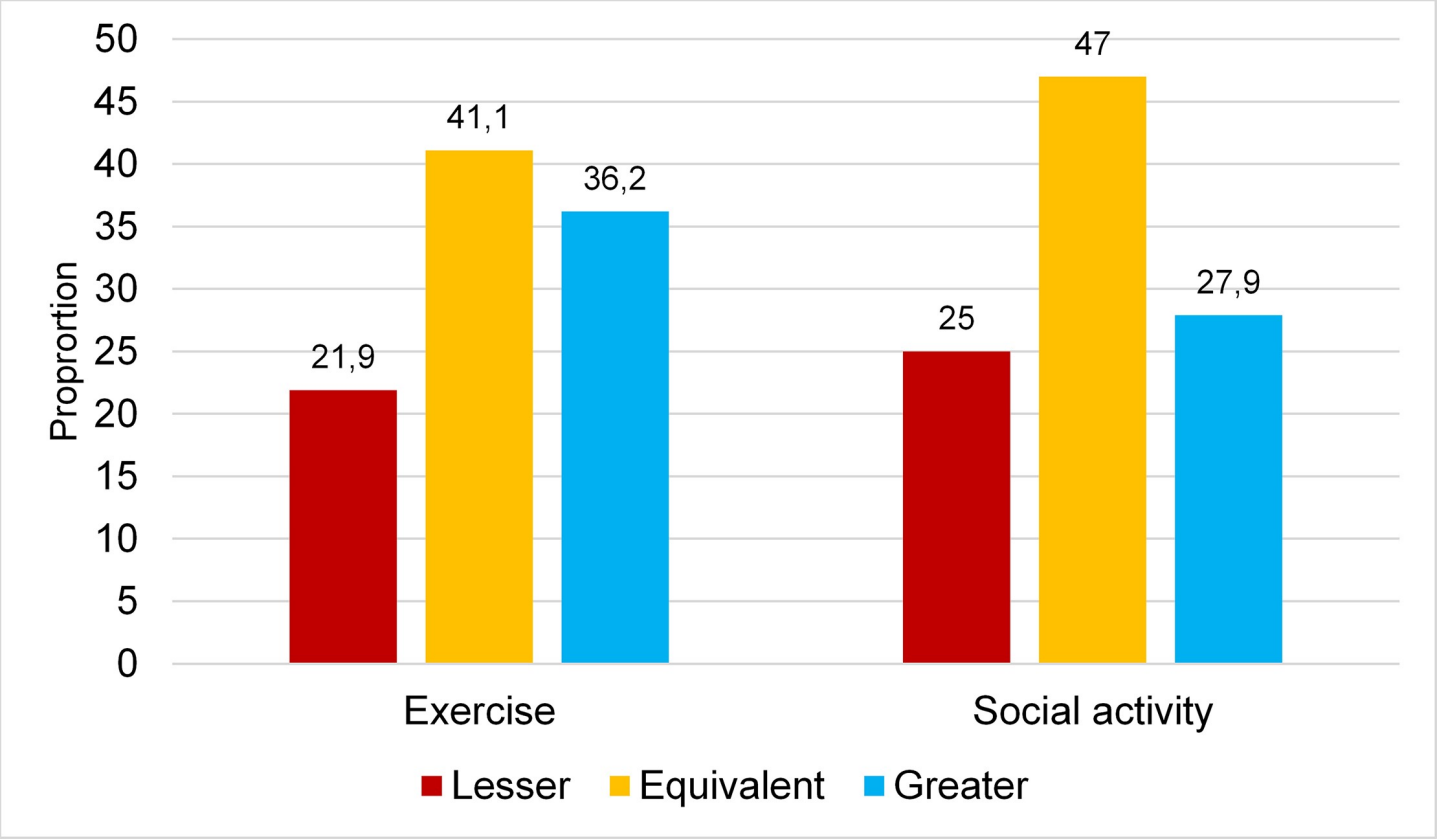
Motivation during activity sessions relative to motivation to go to activity sessions in the exercise and social activity groups.

Motivation during sessions exceeded motivation to go to sessions in 36.2% of cases in the exercise group and 27.9% of cases in the social activity group. Corresponding figures for lower motivation during than before sessions were 21.9% and 25%, respectively (Fig 5).

The cumulative ORs for greater motivation during sessions compared with motivation to go to sessions were 2.39 (95% CI 2.38–2.40) and 1.50 (95% CI 1.32–1.70) in the exercise and social activity groups, respectively. The lack of overlap in CIs indicated a significant difference between groups.

## Discussion

In this study, motivation to participate in high-intensity exercise or in a social activity was generally assessed as high among older people with dementia in nursing homes. No overall difference in motivation during activity sessions was detected between groups. However, motivation during sessions increased over time in the exercise group, whereas it decreased in the social activity group. The motivation scores of some participants fluctuated over the course of the intervention period. Motivation to go to activities was lower in the exercise group and increased over time in both groups, but did not increase to the same extent in the exercise group as in the social activity group. These results did not seem to be influenced by selection bias due to non-attendance. Motivation during activity sessions was higher than motivation to go to sessions in both groups, with a greater difference observed in the exercise group. These results may be important for planning and promoting exercise in older people with dementia in nursing homes, and implicate not to exclude them from high-intensity functional exercise.

This is to our knowledge, the first quantitative study evaluating the motivation to participate in high-intensity functional exercise among older people with dementia in nursing homes. Regardless of apathy, cognitive and physical impairments, high age, and medical conditions, participants’ motivation during activity sessions was high, and good attendance was observed in both groups. Despite the strenuous nature of the exercise program, motivation during the exercise sessions was as high as during the social activity sessions. One might expect that motivation to participate in high-intensity exercise would be lower than motivation to participate in a social group activity in this population, as people with cognitive impairment have been shown to prefer less physically demanding exercise [35]. On the other hand, an individually tailored functional exercise program might be less cognitively demanding than a social activity, and therefore favorable [35]. The participants showed fluctuations in motivation scores between sessions. Forty percent of participants in the exercise group showed at least one change in score of two categories or more, similar to those participating in the less physically demanding social activity. In both groups, such fluctuation may be explained by variations in participants’ daily conditions or the development of new acute medical conditions, which are common in this population [51].

The positive time trend of motivation during the exercise period may be explained by improvements in ADLs and balance in the exercise group over the course of the intervention period [12]. In interviews, participants indicated that they experienced improvements in mental and bodily strength due to the exercise [52]. The exercise program was individually tailored and performed in small groups with the same leaders and participants each time, which might have increased motivation by routine and familiarization. These exercise properties and effects, as well as, the fact that 70% were previous exercisers might imply high self-efficacy for exercise, which has been shown to have an especially critical influence on motivation [23,27,53]. Self-efficacy is affected by, for example, outcome expectations, reliable encouragement, skill training, role models, and mastery experiences, which, in turn, can affect effort, persistence, and achievement [27]. Self-efficacy can increase during exercise in older people with [54] and without dementia, and this increase has been associated with exercise adherence [55]. The aspects of experienced improvement and familiarization can also be interpreted according to the self-determination theory (SDT) [25], which describes motivation in different fields, including physical activity [26]. The basic assumption of the SDT is that human beings have essential needs of autonomy, competence, and relatedness for optimal functioning [25]. Motivation can be improved by taking these basic needs into account. Competence is described as handling the environment in the best possible way, as well as having opportunities to practice and express such capacity. By successfully managing the functional exercises, as well as perceiving improvements in physical function, the study participants might have gained competence satisfaction. Additionally, training in a group of people with competent leaders may have given rise to feelings of relatedness, which is an important factor to consider in this study population, of which more than half experienced loneliness.

Reasons for significantly lower motivation to go to activity sessions than during sessions, especially in the exercise group, might have included anxiety about upcoming events [30], as well as apathy and low initiative [29], all of which are experienced by people with dementia. The exercise activity was supposed to be more physically demanding than the social activity, and the difference in motivation to go to sessions between groups might be explained by anxiety and unease, which have been described as barriers to physical activity [56]. These barriers might not have been equally decisive in the group attending seated social activity sessions. As motivation seems to increase during exercise and over time, the exertion of extra effort to encourage people with dementia to join exercise activities is important. The results from this study and previous studies show the importance of offering personalized exercise for people with dementia [36]. It is particularly emphasized that staff need to know the participants’ past experiences so that they “know what makes them tick” when motivating cognitively impaired older adults to exercise [57]. Addressing people’s positive emotions, bodily memories, and past experience and to use gentle persuasive approach might be effective ways to increase motivation to exercise in people with impaired cognitive function.

In our experience, activity leaders can and often do act as vicarious external motivators, as low initiative and lack of interest in activities are common in people with dementia. Such motivators might be more important at the beginning of an exercise period, when motivation is lower, as in this study. All activity leaders in this study were PTs or OTs/OT assistants experienced in working with people with cognitive impairment, which might have positively affected the results in both groups. In interviews, exercise participants in the UMDEX study indicated that they experienced the exercise as achievable, despite perceiving it as challenging, because it was supervised by skilled leaders [52]. This finding is in line with the results of another study, in which instructors’ competence was found to be an important facilitator for people with dementia [54]. Activity leaders can be important for relatedness and competence. Furthermore, the presence of professional exercise instructors in nursing homes was associated significantly with exercise adherence, duration, and frequency in a previous study [58]. In a focus-group study, PTs in the UMDEX study expressed the importance of building relationships and trust through verbal and non-verbal communication. A learning process how to meet the unique participant’s needs occurred over time during the exercise period [59]. Additionally, the participants in the present study were dependent in ADLs and needed support from another person to be able to join the activities. The significance of help has been described previously [60]. In our study, the activity leaders and staff helped the participants to the sessions when needed, which probably contributed to high attendance and motivation. In a clinical setting, taking participants’ daily routines and needs into account and individualizing factors, such as, when exercise sessions take place are important to increase the motivation to exercise. To reduce sedentary behavior and to maintain functional ability it is important to provide physical activity and exercise in nursing homes. However, despite recent recommendations of exercise [61], most psychogeriatric and somatic nursing home residents spend their day inactive in lying or sitting positions, according to a large observation study [62]. In order to provide best care, it may be most effective that nursing home management are responsible for organizing physical activity and exercise sessions.

A limitation of this study was that the participants did not self-assess their motivation. Because of the participants’ reduced cognition, activity leaders assessed their motivation and eagerness to participate in the activities. The people who most often helped participants to the activity sessions (nursing home staff and activity leaders) assessed participants’ motivation to go to sessions. Another limitation is that the validity and reliability of the motivation scales used have not been tested, which implies that the results must be interpreted with some caution. However, the research group deemed the scales to have good face validity [63], and the prerequisites for a high degree of reliability, as they includes five interchangeable categories that have been described in detail. The scales were well known to the activity leaders in advance [63]. Another factor to consider is that people who tend to volunteer for exercise studies are already motivated to exercise, although how this transfers to people with dementia is more uncertain. In this study, the participants agreed to take part in an activity, either exercise or social. The participation of an old population with dementia and comorbidity is a strength in this study. Another strength includes the capturing of repeated measures over a long intervention period and the statistical method used. Ordinal regression enabled examination of the motivation scales in their original form. Further strengths were the collection of reasons for non-attendance, which enabled the investigation of selection bias.

## Conclusions and implications

Among older people with dementia living in nursing homes, with high prevalence of medical conditions and functional limitations, motivation to participate in a high-intensity exercise program seems to be high and not to differ from the motivation to participate in a less physically demanding social activity. Motivation during exercise sessions can be higher than motivation to go to sessions and motivation can increase over time. Consequently, the promotion of strategies to encourage people with dementia to join exercise groups is of great importance. Furthermore, more knowledge about strategies to overcome low motivation is needed.

## Supporting information

S1 Appendix (DOCX)

S1 File (SAV)

S2 File (SAV)
