# Supplementary material for: Motivation to participate in high-intensity functional exercise compared with a social activity in older people with dementia in nursing homes
Source: PLoS One. 2018 Nov 14;13(11):e0206899. doi: 10.1371/journal.pone.0206899 (PMC6235314; doi:10.1371/journal.pone.0206899)
Supplement: S1 Appendix — (DOCX) [file pone.0206899.s001.docx]

**Investigation of selection bias due to loss of participants**

Hypothetically, increased motivation during sessions over time in the exercise group could be a result of increasing non-attendance, due to low motivation, and decreased motivation during sessions in the social activity group could be due to a decrease in non-attendance because of low motivation. The rates of non-attendance due to low motivation in the first 5 sessions were 8.4% in the exercise group and 4.5% in the social activity group, and differed throughout the intervention period in both groups (Fig 2).

The study data may also be affected by selection bias if the odds of session non-attendance associated with low motivation to go to sessions differed between the exercise and social activity groups. The odds of not attending a session were three and four times as high in the exercise and social activity groups, respectively [OR 3.1 (95% CI 1.7–5.7) and OR 4.0 (95% CI 2.3–7.0)] when individuals had no motivation to go to sessions. The OR for non-attendance in the exercise group relative to the social activity group was 1.3 (95% CI 0.6–2.8).
